# Supplementary material for: Structural Protein Analysis of Driver Gene Mutations in Conjunctival Melanoma
Source: Genes (Basel). 2021 Oct 15;12(10):1625. doi: 10.3390/genes12101625 (PMC8535873; doi:10.3390/genes12101625)
Supplement: Supplementary file 1 [file genes-12-01625-s001.zip › genes-1400919-supplementary.pdf]

Figure S1. **BRAF's amino acid sequence.** The protein sequence below is represented in FASTA form and represents BRAF's canonical sequence. The protein contains 766 amino acid residues.

```
>sp|P15056|BRAF_HUMAN Serine/threonine-protein kinase B-raf OS=Homo
sapiens OX=9606 GN=BRAF PE=1 SV=4
MAALSGGGGGGAEPGQALFNGDMEPEAGAGAGAAASSAADPAIPEEVWNIKQMIKLTQEHIEALLDKFGGEHN
PPSIYLEAYEEYTSKLDALQQREQQLLESLGNGTDFSVSSSASMDTVTSSSSSSLSVLPSSLSVFQNPTDVAR
SNPKSPQKPIVRVFLPNKQRTVVPARCGVTVRDSLKKALMMRGLIPECCAVYRIQDGEKKPIGWDTDISWLTG
EELHVEVLENVPLTTHNFVRKTFFTLAFCDFCRKLLFQGFRCQTCGYKFHQRCSTEVPLMCVNYDQLDLLFVS
KFFEHHPIPQEEASLAETALTSGSSPSAPASDSIGPQILTSPSPSKSIPIPQPFRPADEDHRNQFGQRDRSSS
APNVHINTIEPVNIDDLIRDQGFRGDGGSTTGLSATPPASLPGSLTNVKALQKSPGPQRERKSSSSSEDRNRM
KTLGRRDSSDDWEIPDGQITVGQRIGSGSFGTVYKGKWHGDVAVKMLNVTAPTPQQLQAFKNEVGVLRKTRHV
NILLFMGYSTKPQLAIVTQWCEGSSLYHHLHIIETKFEMIKLIDIARQTAQGMDYLHAKSIIHRDLKSNNIFL
HEDLTVKIGDFGLATVKSRWSGSHQFEQLSGSILWMAPEVIRMQDKNPYSFQSDVYAFGIVLYELMTGQLPYS
NINNRDQIIFMVGRGYLSPDLSKVRSNCPKAMKRLMAECLKKKRDERPLFPQILASIELLARSLPKIHRSASE
PSLNRAGFQTEDFSLYACASPKTPIQAGGYGAFPVH
```

Figure S2. **NRas's amino acid sequence.** The protein sequence below is represented in FASTA form and represents NRas'scanonical sequence. The protein contains 189 amino acid residues.

```
>sp|P01111|RASN_HUMAN GTPase NRas OS=Homo sapiens OX=9606 GN=NRAS PE=1
SV=1
MTEYKLVVVGAGGVGKSALTIQLIQNHFVDEYDPTIEDSYRKQVVIDGETCLLDILDTAGQEEYSAMRDQYMR
TGEGFLCVFAINNSKSFADINLYREQIKRVKDSDDVPMVLVGNKCDLPTRTVDTKQAHELAKSYGIPFIETSA
KTRQGVEDAFYTLVREIRQYRMKKLNSSDDGTQGCMGLPCVVM
```

Figure S3. **C-Kit's amino acid sequence.** The protein sequence below is represented in FASTA form and represents c-Kit's canonical sequence. The protein contains 976 amino acid residues.

```
>sp|P10721|KIT_HUMAN Mast/stem cell growth factor receptor Kit OS=Homo
sapiens OX=9606 GN=KIT PE=1 SV=1
MRGARGAWDFLCVLLLLLRVQTGSSQPSVSPGEPSPPSIHPGKSDLIVRVGDEIRLLCTDPGFVKWTFEILDE
TNENKQNEWITEKAEATNTGKYTCTNKHGLSNSIYVFVRDPAKLFLVDRSLYGKEDNDTLVRCPLTDPEVTNY
SLKGCQGKPLPKDLRFIPDPKAGIMIKSVKRAYHRLCLHCSVDQEGKSVLSEKFILKVRPAFKAVPVVSVSKA
SYLLREGEEFTVTCTIKDVSSSVYSTWKRENSQTKLQEKYNSWHHGDFNYERQATLTISSARVNDSGVFMCYA
NNTFGSANVTTTLEVVDKGFINIFPMINTTVFVNDGENVDLIVEYEAFPKPEHQQWIYMNRTFTDKWEDYPKS
ENESNIRYVSELHLTRLKGTEGGTYTFLVSNSDVNAAIAFNVYVNTKPEILTYDRLVNGMLQCVAAGFPEPTI
DWYFCPGTEQRCSASVLPVDVQTLNSSGPPFGKLVVQSSIDSSAFKHNGTVECKAYNDVGKTSAYFNFAFKGN
NKEQIHPHTLFTPLLIGFVIVAGMMCIIVMILTYKYLQKPMYEVQWKVVEEINGNNYVYIDPTQLPYDHKWEF
PRNRLSFGKTLGAGAFGKVVEATAYGLIKSDAAMTVAVKMLKPSAHLTEREALMSELKVLSYLGNHMNIVNLL
GACTIGGPTLVITEYCCYGDLLNFLRRKRDSFICSKQEDHAEAALYKNLLHSKESSCSDSTNEYMDMKPGVSY
VVPTKADKRRSVRIGSYIERDVTPAIMEDDELALDLEDLLSFSYQVAKGMAFLASKNCIHRDLAARNILLTHG
RITKICDFGLARDIKNDSNYVVKGNARLPVKWMAPESIFNCVYTFESDVWSYGIFLWELFSLGSSPYPGMPVD
SKFYKMIKEGFRMLSPEHAPAEMYDIMKTCWDADPLKRPTFKQIVQLIEKQISESTNHIYSNLANCSPNRQKP
VVDHSVRINSVGSTASSSQPLLVHDDV
```

Figure S4. **NF1's amino acid sequence.** The protein sequence below is represented in FASTA form and represents NF1's canonical sequence. The protein contains 2,839 amino acid residues.

```
>sp|P21359|NF1_HUMAN Neurofibromin OS=Homo sapiens OX=9606 GN=NF1 PE=1
SV=2
MAAHRPVEWVQAVVSRFDEQLPIKTGQQNTHTKVSTEHNKECLINISKYKFSLVISGLTTILKNVNNMRIFGE
AAEKNLYLSQLIILDTLEKCLAGQPKDTMRLDETMLVKQLLPEICHFLHTCREGNQHAAELRNSASGVLFSLS
CNNFNAVFSRISTRLQELTVCSEDNVDVHDIELLQYINVDCAKLKRLLKETAFKFKALKKVAQLAVINSLEKA
FWNWVENYPDEFTKLYQIPQTDMAECAEKLFDLVDGFAESTKRKAAVWPLQIILLILCPEIIQDISKDVVDEN
NMNKKLFLDSLRKALAGHGGSRQLTESAAIACVKLCKASTYINWEDNSVIFLLVQSMVVDLKNLLFNPSKPFS
RGSQPADVDLMIDCLVSCFRISPHNNQHFKICLAQNSPSTFHYVLVNSLHRIITNSALDWWPKIDAVYCHSVE
LRNMFGETLHKAVQGCGAHPAIRMAPSLTFKEKVTSLKFKEKPTDLETRSYKYLLLSMVKLIHADPKLLLCNP
RKQGPETQGSTAELITGLVQLVPQSHMPEIAQEAMEALLVLHQLDSIDLWNPDAPVETFWEISSQMLFYICKK
LTSHQMLSSTEILKWLREILICRNKFLLKNKQADRSSCHFLLFYGVGCDIPSSGNTSQMSMDHEELLRTPGAS
LRKGKGNSSMDSAAGCSGTPPICRQAQTKLEVALYMFLWNPDTEAVLVAMSCFRHLCEEADIRCGVDEVSVHN
LLPNYNTFMEFASVSNMMSTGRAALQKRVMALLRRIEHPTAGNTEAWEDTHAKWEQATKLILNYPKAKMEDGQ
AAESLHKTIVKRRMSHVSGGGSIDLSDTDSLQEWINMTGFLCALGGVCLQQRSNSGLATYSPPMGPVSERKGS
MISVMSSEGNADTPVSKFMDRLLSLMVCNHEKVGLQIRTNVKDLVGLELSPALYPMLFNKLKNTISKFFDSQG
QVLLTDTNTQFVEQTIAIMKNLLDNHTEGSSEHLGQASIETMMLNLVRYVRVLGNMVHAIQIKTKLCQLVEVM
MARRDDLSFCQEMKFRNKMVEYLTDWVMGTSNQAADDDVKCLTRDLDQASMEAVVSLLAGLPLQPEEGDGVEL
MEAKSQLFLKYFTLFMNLLNDCSEVEDESAQTGGRKRGMSRRLASLRHCTVLAMSNLLNANVDSGLMHSIGLG
YHKDLQTRATFMEVLTKILQQGTEFDTLAETVLADRFERLVELVTMMGDQGELPIAMALANVVPCSQWDELAR
VLVTLFDSRHLLYQLLWNMFSKEVELADSMQTLFRGNSLASKIMTFCFKVYGATYLQKLLDPLLRIVITSSDW
QHVSFEVDPTRLEPSESLEENQRNLLQMTEKFFHAIISSSSEFPPQLRSVCHCLYQATCHSLLNKATVKEKKE
NKKSVVSQRFPQNSIGAVGSAMFLRFINPAIVSPYEAGILDKKPPPRIERGLKLMSKILQSIANHVLFTKEEH
MRPFNDFVKSNFDAARRFFLDIASDCPTSDAVNHSLSFISDGNVLALHRLLWNNQEKIGQYLSSNRDHKAVGR
RPFDKMATLLAYLGPPEHKPVADTHWSSLNLTSSKFEEFMTRHQVHEKEEFKALKTLSIFYQAGTSKAGNPIF
YYVARRFKTGQINGDLLIYHVLLTLKPYYAKPYEIVVDLTHTGPSNRFKTDFLSKWFVVFPGFAYDNVSAVYI
YNCNSWVREYTKYHERLLTGLKGSKRLVFIDCPGKLAEHIEHEQQKLPAATLALEEDLKVFHNALKLAHKDTK
VSIKVGSTAVQVTSAERTKVLGQSVFLNDIYYASEIEEICLVDENQFTLTIANQGTPLTFMHQECEAIVQSII
HIRTRWELSQPDSIPQHTKIRPKDVPGTLLNIALLNLGSSDPSLRSAAYNLLCALTCTFNLKIEGQLLETSGL
CIPANNTLFIVSISKTLAANEPHLTLEFLEECISGFSKSSIELKHLCLEYMTPWLSNLVRFCKHNDDAKRQRV
TAILDKLITMTINEKQMYPSIQAKIWGSLGQITDLLDVVLDSFIKTSATGGLGSIKAEVMADTAVALASGNVK
LVSSKVIGRMCKIIDKTCLSPTPTLEQHLMWDDIAILARYMLMLSFNNSLDVAAHLPYLFHVVTFLVATGPLS
LRASTHGLVINIIHSLCTCSQLHFSEETKQVLRLSLTEFSLPKFYLLFGISKVKSAAVIAFRSSYRDRSFSPG
SYERETFALTSLETVTEALLEIMEACMRDIPTCKWLDQWTELAQRFAFQYNPSLQPRALVVFGCISKRVSHGQ
IKQIIRILSKALESCLKGPDTYNSQVLIEATVIALTKLQPLLNKDSPLHKALFWVAVAVLQLDEVNLYSAGTA
LLEQNLHTLDSLRIFNDKSPEEVFMAIRNPLEWHCKQMDHFVGLNFNSNFNFALVGHLLKGYRHPSPAIVART
VRILHTLLTLVNKHRNCDKFEVNTQSVAYLAALLTVSEEVRSRCSLKHRKSLLLTDISMENVPMDTYPIHHGD
PSYRTLKETQPWSSPKGSEGYLAATYPTVGQTSPRARKSMSLDMGQPSQANTKKLLGTRKSFDHLISDTKAPK
RQEMESGITTPPKMRRVAETDYEMETQRISSSQQHPHLRKVSVSESNVLLDEEVLTDPKIQALLLTVLATLVK
YTTDEFDQRILYEYLAEASVVFPKVFPVVHNLLDSKINTLLSLCQDPNLLNPIHGIVQSVVYHEESPPQYQTS
YLQSFGFNGLWRFAGPFSKQTQIPDYAELIVKFLDALIDTYLPGIDEETSEESLLTPTSPYPPALQSQLSITA
NLNLSNSMTSLATSQHSPGIDKENVELSPTTGHCNSGRTRHGSASQVQKQRSAGSFKRNSIKKIV
```

Figure S5. **PTEN's amino acid sequence.** The protein sequence below is represented in FASTA form and represents PTEN canonical sequence. The protein contains 403 amino acid residues.

```
>sp|P60484|PTEN_HUMAN Phosphatidylinositol 3,4,5-trisphosphate 3-
phosphatase and dual-specificity protein phosphatase PTEN OS=Homo sapiens
OX=9606 GN=PTEN PE=1 SV=1
MTAIIKEIVSRNKRRYQEDGFDLDLTYIYPNIIAMGFPAERLEGVYRNNIDDVVRFLDSKHKNHYKIYNLCAE
RHYDTAKFNCRVAQYPFEDHNPPQLELIKPFCEDLDQWLSEDDNHVAAIHCKAGKGRTGVMICAYLLHRGKFL
KAQEALDFYGEVRTRDKKGVTIPSQRRYVYYYSYLLKNHLDYRPVALLFHKMMFETIPMFSGGTCNPQFVVCQ
LKVKIYSSNSGPTRREDKFMYFEFPQPLPVCGDIKVEFFHKQNKMLKKDKMFHFWVNTFFIPGPEETSEKVEN
GSLCDQEIDSICSIERADNDKEYLVLTLTKNDLDKANKDKANRYFSPNFKVKLYFTKTVEEPSNPEASSSTSV
TPDVSDNEPDHYRYSDTTDSDPENEPFDEDQHTQITKV
```
